# Vitamin D-*VDR* (vitamin D receptor) alleviates glucose metabolism reprogramming in lipopolysaccharide-induced acute kidney injury

**DOI:** 10.3389/fphys.2023.1083643

**Published:** 2023-02-24

**Authors:** Qing Dai, Hao Zhang, Shiqi Tang, Xueqin Wu, Jianwen Wang, Bin Yi, Jishi Liu, Zhi Li, Qin Liao, Aimei Li, Yan Liu, Wei Zhang

**Affiliations:** ^1^ Department of Nephrology, Third Xiangya Hospital, Central South University, Changsha, China; ^2^ Department of Anesthesiology, Third Xiangya Hospital, Central South University, Changsha, China

**Keywords:** vitamin D, vitamin D receptor, glucose metabolism reprogramming, acute kidney injury, PDHA1 phosphorylation, AMPK pathway

## Abstract

**Background:** Our previous study showed that vitamin D (VD)-vitamin D receptor (*VDR*) plays a nephroprotective role in lipopolysaccharide (LPS)-induced acute kidney injury (AKI). Recently, glucose metabolism reprogramming was reported to be involved in the pathogenesis of AKI.

**Objective:** To investigate the role of VD-*VDR* in glucose metabolism reprogramming in LPS-induced AKI.

**Methods:** We established a model of LPS-induced AKI in *VDR* knockout (*VDR*-KO) mice, renal proximal tubular-specific *VDR*-overexpressing (*VDR*-OE) mice and wild-type C57BL/6 mice. *In vitro*, human proximal tubular epithelial cells (HK-2 cells), *VDR* knockout and *VDR* overexpression HK-2 cell lines were used.

**Results:** Paricalcitol (an active vitamin D analog) or *VDR*-OE reduced lactate concentration, hexokinase activity and PDHA1 phosphorylation (a key step in inhibiting aerobic oxidation) and simultaneously ameliorated renal inflammation, apoptosis and kidney injury in LPS-induced AKI mice, which were more severe in *VDR*-KO mice. In *in vitro* experiments, glucose metabolism reprogramming, inflammation and apoptosis induced by LPS were alleviated by treatment with paricalcitol or dichloroacetate (DCA, an inhibitor of p-*PDHA1*). Moreover, paricalcitol activated the phosphorylation of *AMP*-activated protein kinase (*AMPK*), and an *AMPK* inhibitor partially abolished the protective effect of paricalcitol in LPS-treated HK-2 cells.

**Conclusion:** VD-*VDR* alleviated LPS-induced metabolic reprogramming in the kidneys of AKI mice, which may be attributed to the inactivation of *PDHA1* phosphorylation *via* the *AMPK* pathway.

## 1 Introduction

Acute kidney injury (AKI) is a critical clinical syndrome with high incidence and mortality in hospitalized patients, especially in intensive care unit (ICU) patients, and there are very limited treatment options at hand (Ronco et al., 2019). Sepsis is the most common cause of severe AKI in critically ill patients (Hoste et al., 2015), and its mortality rate is approximately 30% (Bouchard et al., 2015). Lipopolysaccharide (LPS, an endotoxin from the gram-negative bacterial wall), a well-known component that induces sepsis, is widely used in research on sepsis-associated AKI (SA-AKI) (Stasi et al., 2017).

SA-AKI is associated with glomerular and tubular cell damage, mainly due to systemic inflammation, changes in renal hemodynamics, and several other mechanisms (Dellepiane et al., 2016). Recently, the role of metabolic reprogramming in SA-AKI progression has been recognized, and targeting metabolic reprogramming represents a potentially effective therapeutic strategy for the progression of SA-AKI (Toro et al., 2021). Glucose metabolism reprogramming refers to the process of switching the glucose metabolism pathway from oxidative phosphorylation to glycolysis in the presence of sufficient oxygen, during which the activity of hexokinase increases, lactic acid accumulates, and the activity of the pyruvate dehydrogenase complex (*PDH*c) decreases (Vander Heiden et al., 2009; Biswas, 2015; Zhu et al., 2022). It has been found that the metabolism in septic mice induced by cecal ligation and puncture (CLP) and LPS-treated proximal tubule cells changes from oxidative phosphorylation to glycolysis in the presence of sufficient oxygen (Li et al., 2020; Tan et al., 2021). Moreover, the glycolysis induced by LPS-injected mice was associated with decreased renal function (Smith et al., 2014). These findings suggest the involvement of reprogramming glucose metabolism in SA-AKI.

Pyruvate dehydrogenase E1 subunit alpha 1 (*PDHA1*), the key regulatory site of *PDH*c, catalyzes the conversion of pyruvate into acetyl-CoA after it enters mitochondria (Zhou et al., 2001). It regulates the activity of *PDH*c through phosphorylation and dephosphorylation to affect the metabolic flux of glycolysis and the tricarboxylic acid cycle in mitochondria (Holness and Sugden, 2003). Kolobova et al. (2001) confirmed that specific site phosphorylation of *PDHA1* can inhibit *PDH*c activity (Kolobova et al., 2001). It has been reported that the phosphorylation level of *PDHA1* increased in CLP-AKI model mice (Li et al., 2020). Moreover, Mao et al. found that reducing the phosphorylation level of *PDHA1* mitigated LPS-induced endothelial barrier dysfunction (Mao et al., 2022). Therefore, we speculate that inhibition of *PDHA1* phosphorylation may be the target of SA-AKI treatment, but there are no relevant research reports at present.

*VDR* (vitamin D receptor) is highly expressed in the kidney and exerts nephroprotective effects through multiple mechanisms. In our previous studies, we demonstrated that 1, 2, 5(OH)2D3 (active vitamin D) or its active analogs can exert renal protection in lipopolysaccharide (LPS)-induced acute kidney injury by activating *VDR* (Du et al., 2019). Current studies have shown that 1,25(OH)2D3 treatment can alleviate the abnormal reprogramming of glucose metabolism in breast cancer (Santos et al., 2018), and it can also promote the transformation of dendritic cells and HEK293T cells to aerobic oxidation (Ferreira et al., 2015; Santos et al., 2017). However, whether VD-*VDR* can alleviate SA-AKI by regulating glucose metabolism reprogramming is unclear. In this work, we aimed to explore the role of VD-*VDR* in glucose metabolism reprogramming in an AKI model induced by LPS and elucidate its potential regulatory mechanism.

## 2 Materials and methods

### 2.1 Animal experiment

Wild type male C57BL/6 mice were purchased from Slyke jingda Biotechnology Company (CertificateSCXK 2016-0002; Hunan, China). The *VDR* knockout (*VDR*-KO), renal proximal tubular specific *VDR* overexpressing (*VDR*-OE) mice and littermates were constructed in cooperation with the Model Animal Research Center of Nanjing University. All experimental mice were fed under SPF conditions, and the experimental protocols were approved by the Laboratory Animal Ethics Committee of Central South University.

To induce AKI, 8-week-old male mice received intraperitoneal injections of either PBS (WT group) or 20 mg/kg LPS (O111:B4, L2630, Sigma Aldrich, LPS group) for one dose. To investigate the effect of *VDR* activation on LPS-induced AKI, mice were injected intraperitoneally with paricalcitol (an activated vitamin D analog, a present from professor Yan Chun Li, Chicago university, 0.2 μg/kg/day for wild-type mice (Du et al., 2019) and 0.1 μg/kg/day for *VDR*-OE mice (Jiang et al., 2021), respectively, 1 week before LPS injection) or the same volume of solvent. Finally, wild type male C57BL/6 mice were randomly divided into WT, WT + P, LPS group and LPS + P group; *VDR*-KO mice and their littermates were randomly divided into WT, KO, LPS, KO + LPS groups; and OE mice and their littermates were randomly divided into WT, OE, LPS, and OE + LPS groups. All mice were sacrificed 24 h after LPS administration. Their blood and kidneys were collected for subsequent experimental analysis.

### 2.2 Cell culture and treatment

Human proximal tubular epithelial cells (HK-2 cells) and their *VDR* knockout (*VDR*-KO) cell lines were used in this study, provided by the Institute of Nephrology, Central South University. HK-2 cells were transfected with VDR plasmid and blank plasmid using Lipofectamine 2000, and cultured in F12 (1:1) DMEM supplemented with 10% fetal bovine serum. Cells were seeded in six-well plates at a rate of 5 × 10^4^ cells/well. After 24 h of incubation at 37°C, 5% CO_2_, the cell cultures were supplemented with paricalcitol (200 nM for HK-2cells, 100 nM for *VDR*-OE cells) for 24 h, and then treated with LPS (1 μg/mL) for a further 16–24 h to harvest cells for follow-up experiments. In some experiments, DCA (5 mM, 2 h pretreatment, 347795, Sigma Aldrich), Compound C (10 μM, 1h pretreatment, HY-13418, MedChemExpress) were used.

### 2.3 Measurement of BUN, Cr, lactate, and hexokinase activity

Blood and renal tissues were collected for biochemical analysis. BUN and creatinine levels in serum, and lactate levels, hexokinase activity in renal tissues were measured using the corresponding detection kits in accordance with the manufacturer’s instructions. All these ELISA kits purchased from Nanjing JianCheng Bioengineering Institute (Nanjing, China). In addition, lysate of HK-2 cells was also collected for detection of lactate levels (KTB1100, Abbkine).

### 2.4 Mitochondrial morphology observation by electron microscopy

Tissues were embedded and cut into 50–100 nm ultrathin sections by an ultramicrotome and a diamond knife. Then, they were double stained with 3% uranyl acetate and lead nitrate and examined with a Hitachi HT- 7700 electron microscope.

### 2.5 Renal tissue histopathological and immunofluorescence staining

Briefly, isolated mouse kidney tissue was fixed immediately with formalin and embedded in paraffin, then, the tissue was cut into 5 μm thick sections for hematoxylin-eosin (H&E) staining, TUNEL fluorescent staining, F4/80 fluorescent staining, p-*PDHA1* fluorescent staining and *PDHA1* fluorescent staining. The intensities of p-*PDHA1* and *PDHA1* in the photos were detected by Image J software.

### 2.6 Western blot analysis

After extraction of tissue and cellular protein, protein was separated by SDS/PAGE and electro-transferred to PVDF membranes. The resulting membranes were blocked with 0.1% (w/v) BSA solution on a shaker for 1 h. Then, they were incubated with primary antibodies at 4°C overnight. The antibodies including: *VDR* (ab109234, Abcam), cleaved caspase-3 (ab49822, ab214430, Abcam), bcl2 (226593-1-AP, ProteinTech), *PDHA1* (sc-377092, Santa Curz Biotechnology), p-*PDHA1* (ab177461, Abcam), p-*AMPK* (2535, Cell Signaling Technology), *AMPK* (2532, Cell Signaling Technology), β-actin (20536-1-AP, ProteinTech), α-tublin (AF7010, Affinity). After that, the membrane was incubated with the fluorescent secondary antibody for 1 h before three times with TBST. Finally, the protein expression levels were visualized by the Image Studio software and band intensities were quantified with Image J gel analysis software.

### 2.7 Real-time quantitative PCR

Total RNA was extracted from the renal cortex and cell using the corresponding detection kits in accordance with the manufacturer’s instructions. cDNA was synthesized using a reverse transcription kit. Real-time quantitative PCR was performed using SYBR Green PCR Master Mix on a Roche Light Cycler 480 system. PCR primer sequences are shown in Table 1 (Chen et al., 2013; Li et al., 2022). The relative mRNA levels were calculated using the 2^−ΔΔCT^ formula.

**TABLE 1:** Primers and oligonucleotide probe sequences used in the study.

	Mouse	Human
IL6	F: ATA​GTC​CTT​CCT​ACC​CCA​ATT​TCC	F: GAC​AGC​CAC​TCA​CCT​CTT​CA
	R: CTG​ACC​ACA​GTG​AGG​AAT​GTC​CAC	R: GCC​TCT​TTG​CTG​CTT​TCA​CA
MCP1	F: CAC​TCA​CCT​GCT​GCT​ACT​CA	F: AGC​AGC​AAG​TGT​CCC​AAA​GA
	R: CTT​CTT​GGG​GTC​AGC​ACA​GA	R: CGG​AGT​TTG​GGT​TTG​CTT​GT
ACTIN	F: CAT​TGC​TGA​CAG​GAT​GCA​GAA​GG	F: CAT​GTA​CGT​TGC​TAT​CCA​GGC
	R: TGC​TGG​AAG​GTG​GAC​AGT​GAG​G	R: CTC​CTT​AAT​GTC​ACG​CAC​GAT

### 2.8 Oxygen consumption rate (OCR)

Oxygen consumption rate (OCR) was measured using the SeahorseXF96 Extracellular Flux Analyzer (Seahorse Bioscience, North Billerica, MA, United States). HK-2 cells were seeded into 96-well cell culture plate at a density of 1.5 × 104 cells. When the cell confluence was about 90%, cells were washed twice with assay medium (49.5 mL basal medium, 500 μL sodium pyruvate and basal medium) and incubated in a non-CO_2_ incubator for 40–60 min, OCR was measured. The working fluid concentration was as follows: oligomycin (1 μM), FCCP (2.5 μM), rotenone and antimycin A (1 μM).

### 2.9 Statistical analysis

All data were presented as means ± SD. Statistical comparisons were carried out using unpaired two-tailed Student’s *t*-test or one-way analysis of variance (ANOVA) as appropriate. Statistical significance was defined as *p* < 0.05.

## 3 Results

### 3.1 *VDR* deficiency aggravated LPS-induced renal injury and glucose metabolism reprogramming

**FIGURE 1:**
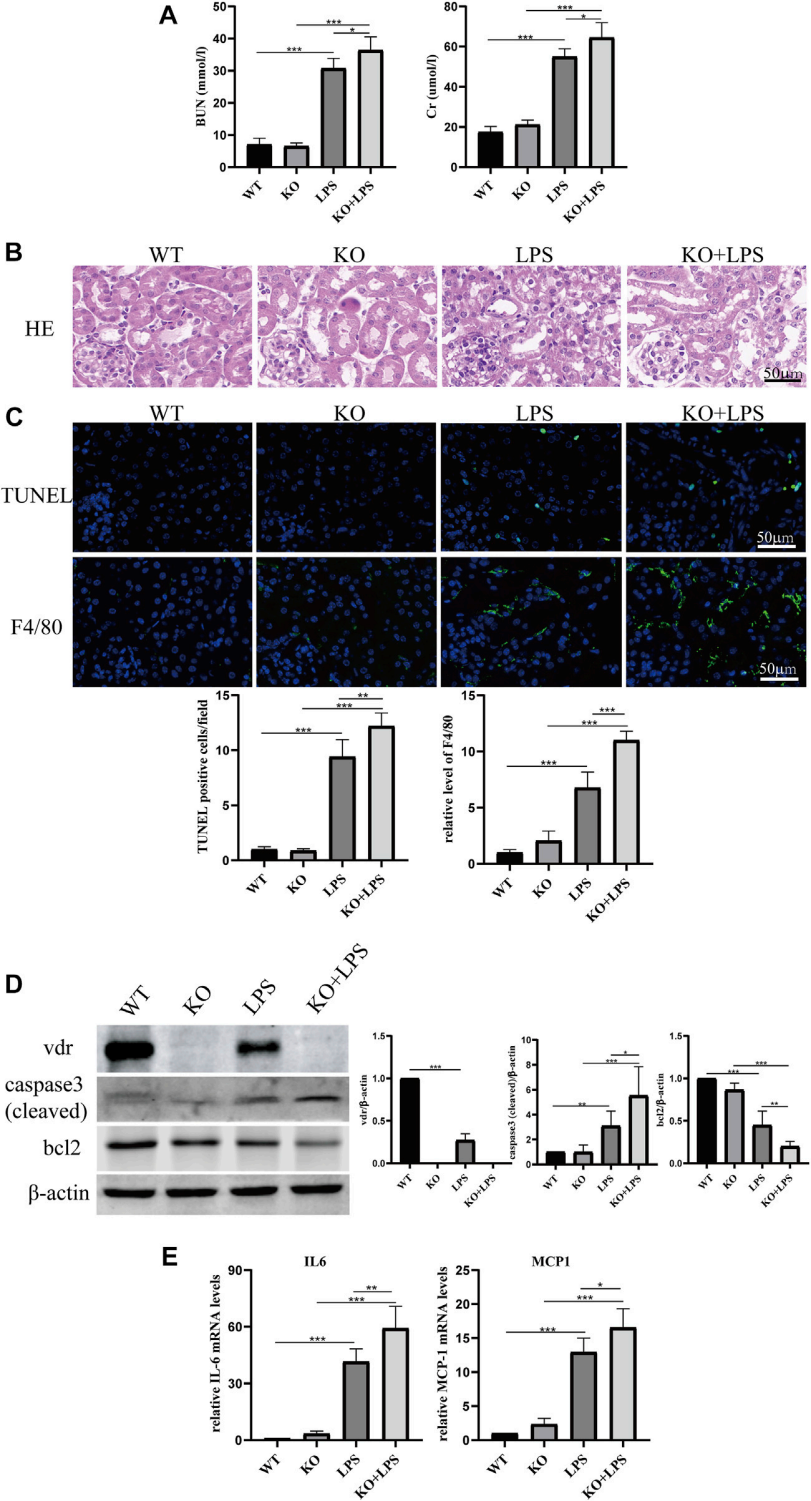
Effects of *VDR* deletion on LPS-induced AKI mice. **(A)** Serum concentrations of BUN and Cr at 24 h after LPS administration. **(B)** H&E staining of kidney sections. **(C)** Immunofluorescence analysis and its quantitative analysis of TUNEL (top) and F4/80 (bottom) of kidney sections. **(D)** Western blot analysis (left) and densitometric quantitation (right) of *VDR*, bcl2 and cleaved caspase3 was performed in the four groups of mice. **(E)** Real-time RT-PCR quantification of IL-6 and MCP1 in the renal cortex of the four groups of mice. *p < 0.05; **p < 0.01; ***p < 0.001. *VDR*, vitamin D receptor.

As shown in Figure 1A, LPS-induced loss of renal function featured with elevated BUN and creatinine levels, and the levels were further increased in *VDR*-KO mice. HE staining revealed severe tubular dilation, cell shedding and brush-border disruption in the kidney cortex after LPS injection, and these pathological lesions were more serious in *VDR*-KO mice than in their WT littermates (Figure 1B). Additionally, immunofluorescence analysis of TUNEL and F4/80 (a marker of macrophage infiltration) in kidney sections showed that elevated tubular cell apoptosis and interstitial inflammatory infiltrate in LPS-treated mice were aggravated in the KO + LPS group (Figure 1C). In addition, compared with the LPS group, the KO + LPS group showed more robust caspase-3 (cleaved) activation, weaker *bcl2* in western blot analysis and higher mRNA expression of *IL-6* and *MCP-1* by real-time RT‒PCR (Figures 1D, E). These results are consistent with those of our previous study (Du et al., 2019).

**FIGURE 2:**
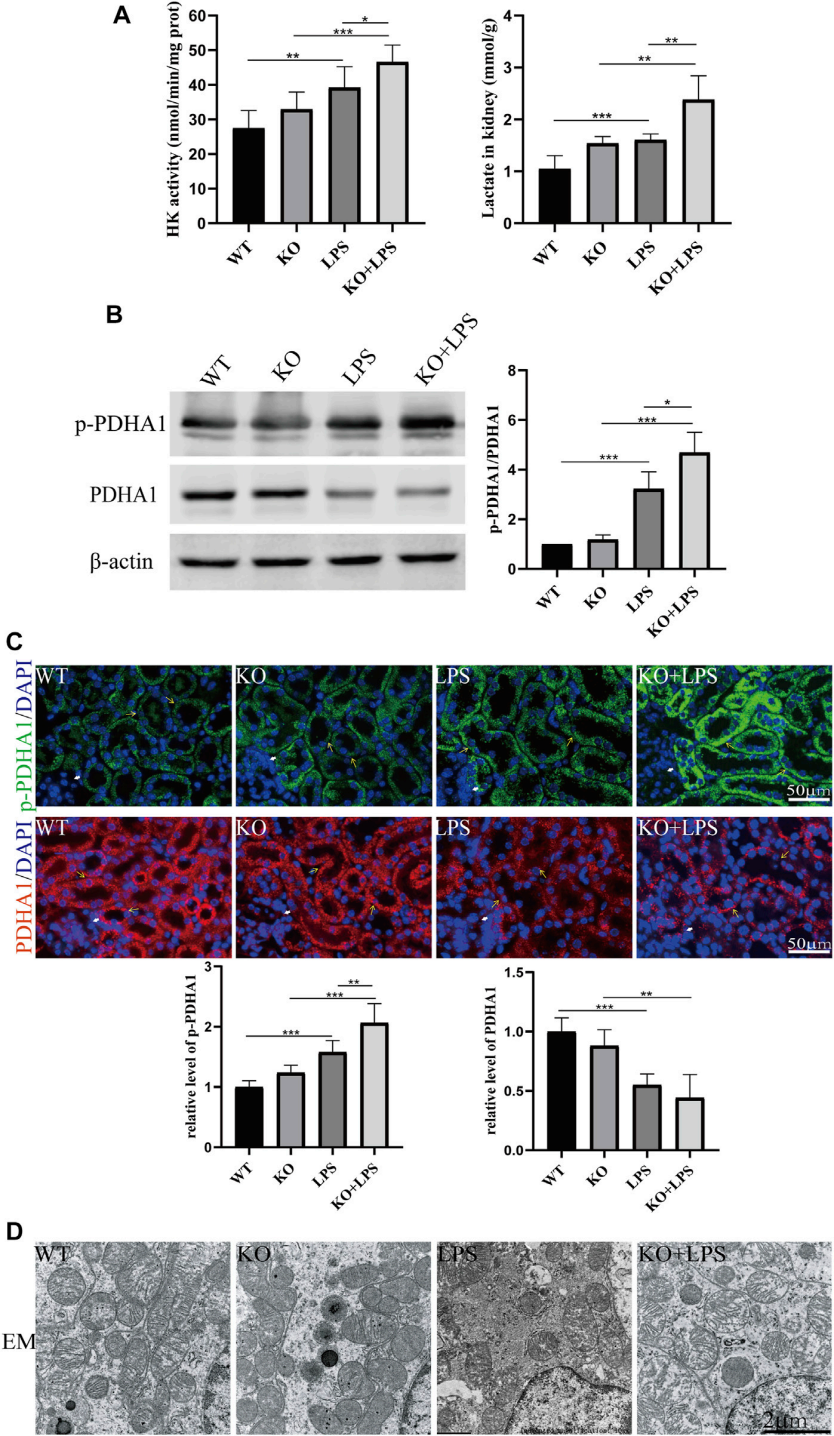
*VDR* deletion aggravated the abnormal glycolysis of LPS-induced AKI mice. **(A)** Renal lactate content and hexokinase activity of the four groups. **(B)** Western blot analysis (left) and densitometric quantitation (right) of *PDHA1* and p-*PDHA1* and was performed in the four groups of mice. **(C)** Immunofluorescence analysis and its quantitative analysis of p-*PDHA1* (green) and *PDHA1* (red) of kidney sections. White arrow: glomerulus; yellow arrow: renal tubules. **(D)** Images of mitochondrial injury of proximal tubule epithelial cells of mice. *p < 0.05; **p < 0.01; ***p < 0.001.

More importantly, we found that the lactate concentration and hexokinase activity in renal homogenate were increased after LPS injection at 24 h and further increased in *VDR*-KO mice (Figure 2A). Western blot and immunofluorescence analyses showed that the protein expression level of p-*PDHA1*/*PDHA1* (a key catalytic enzyme that adjusts the tricarboxylic acid cycle and oxidative phosphorylation during glycolysis through phosphorylation and dephosphorylation) was increased in LPS-injected mice, and *VDR*-KO mice showed a further increase in the *p-PDHA1*/*PDHA1* ratio in renal tissue (Figures 2B, C). Since mitochondria are the main site of aerobic oxidation, we observed morphological changes in mitochondria through transmission electron microscopy, and the results showed that LPS-treated kidney tissue had more swollen mitochondria and a reduced number of cristae with a more lamellar phenotype, and these characteristics were significantly exacerbated in *VDR*-KO mice (Figure 2D). These results confirm that *VDR* deficiency aggravated LPS-induced renal injury and glucose metabolism reprogramming.

### 3.2 *VDR* overexpression alleviates renal injury and glucose metabolism reprogramming in LPS-induced AKI

**FIGURE 3:**
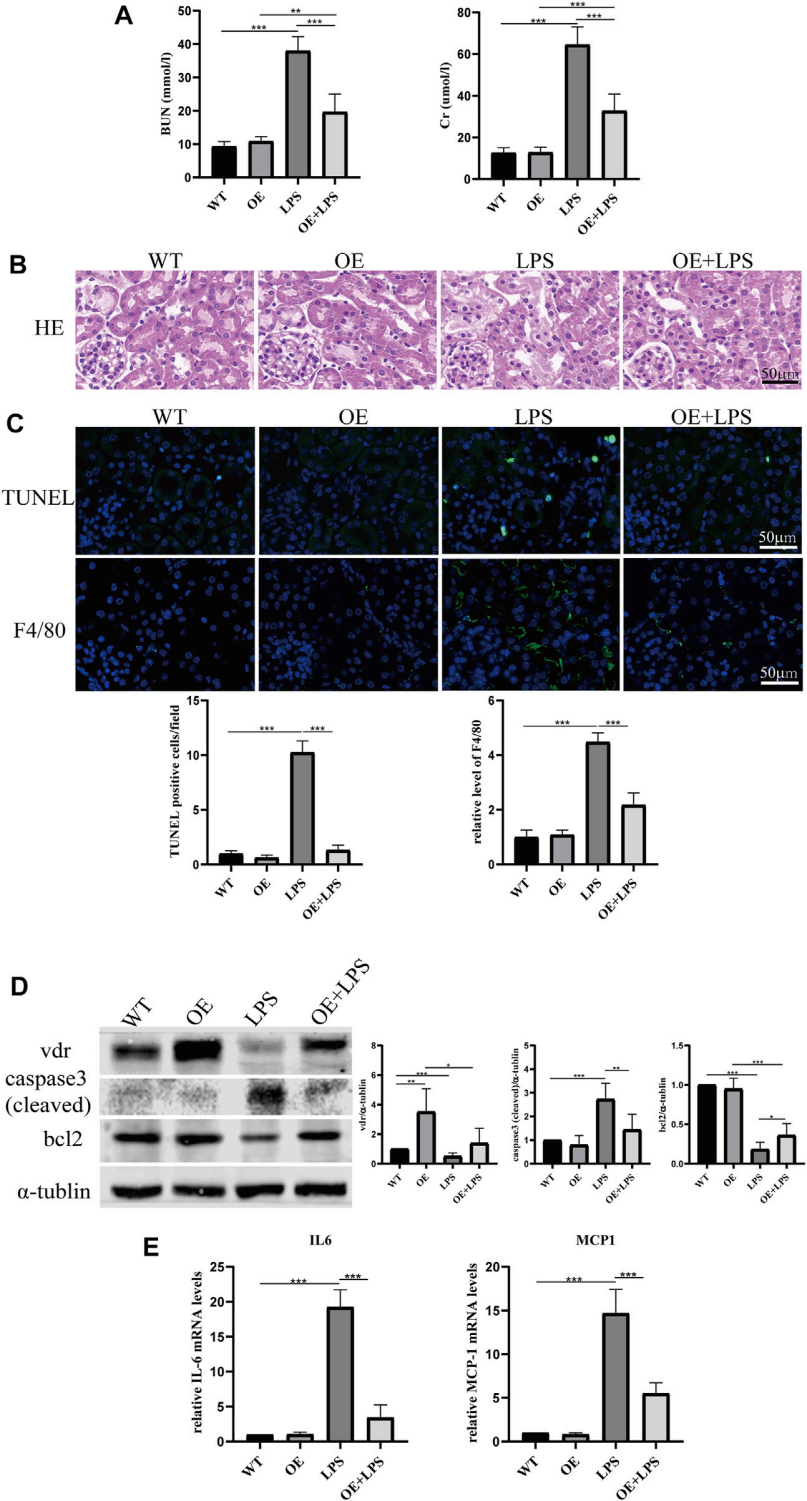
Effects of *VDR* overexpression on LPS-induced AKI mice. **(A)** Serum concentrations of BUN and Cr at 24 h after LPS administration. **(B)** H&E staining of kidney sections. **(C)** Immunofluorescence analysis and its quantitative analysis of TUNEL (top) and F4/80 (bottom) of kidney sections. **(D)** Western blot analysis (left) and densitometric quantitation (right) of *VDR*, bcl2 and cleaved caspase3 was performed in the four groups of mice. **(E)** Real-time RT-PCR quantification of IL-6 and MCP1 in the renal cortex of the four groups of mice. *p < 0.05; **p < 0.01; ***p < 0.001.

**FIGURE 4:**
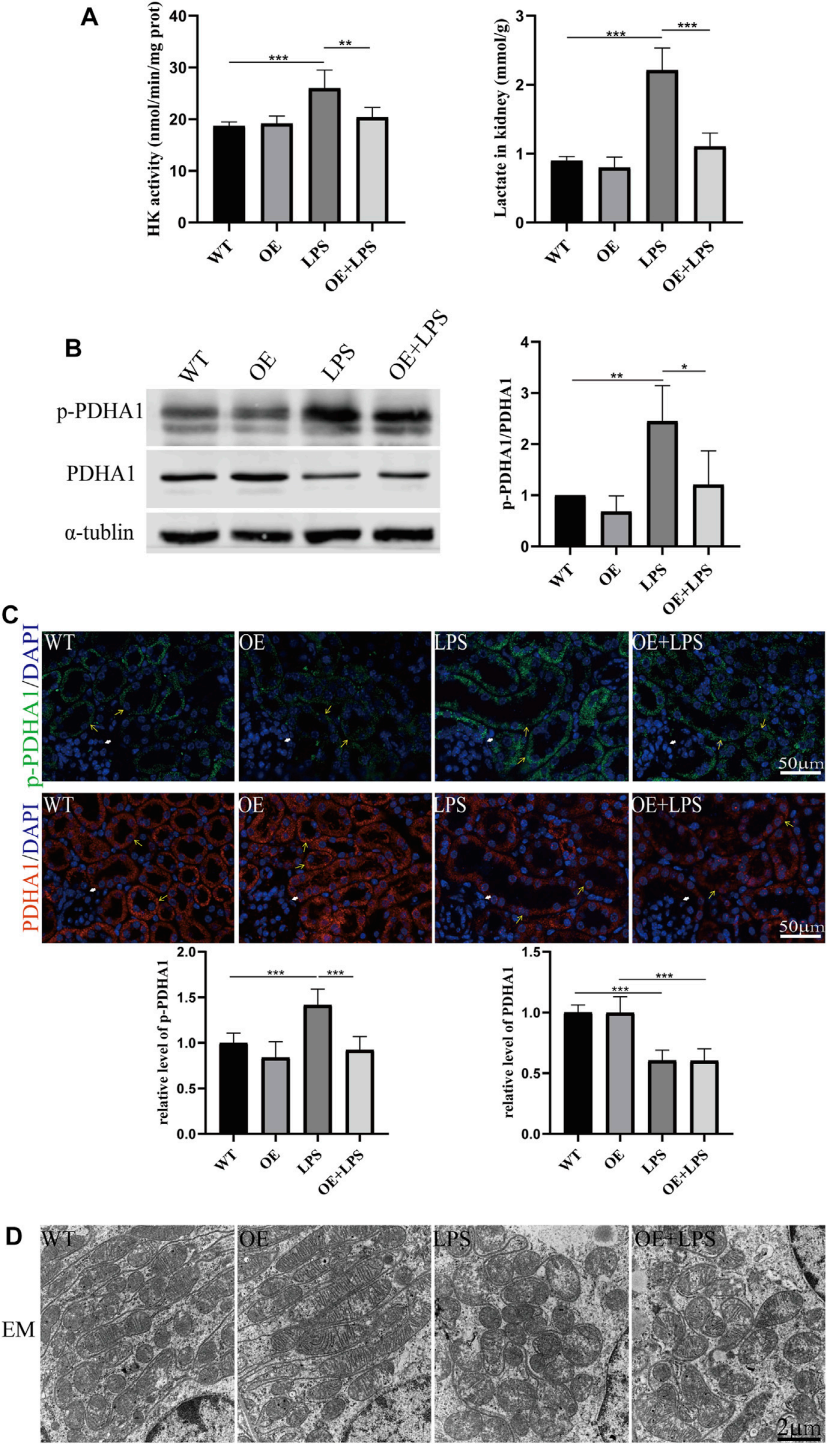
*VDR* overexpression lightened the abnormal glycolysis of LPS-induced AKI mice. **(A)** Renal lactate content and hexokinase activity of the four groups. **(B)** Western blot analysis (left) and densitometric quantitation (right) of *PDHA1* and p-*PDHA1* and was performed in the four groups of mice. **(C)** Immunofluorescence analysis and its quantitative analysis of p-*PDHA1* (green) and *PDHA1* (red) of kidney sections. White arrow: glomerulus; yellow arrow: renal tubules. **(D)** Images of mitochondrial injury of proximal tubule epithelial cells from the four groups of mice. *p < 0.05; **p < 0.01; ***p < 0.001.

To further confirm the role of *VDR* in glucose metabolism reprogramming, we constructed an LPS-induced AKI model in transgenic mice with renal proximal tubular-specific *VDR*-overexpressing (*VDR*-OE). As expected, compared with WT littermates treated with the same dose of LPS, renal function, kidney cortex pathological lesions, tubular cell apoptosis and interstitial inflammation were partially ameliorated in *VDR*-OE mice (Figure 3). Consistently, the increased lactate concentration and hexokinase activity in renal homogenate were lowered by overexpression of *VDR* (Figure 4A), and the ratio of renal p*-PDHA1*/*PDHA1* expression was also significantly decreased in *VDR*-OE mice (Figures 4B, C). Electron microscopy showed that the swelling of mitochondria in *VDR*-OE mice treated with LPS was improved compared to their WT littermates treated with LPS (Figure 4D). These results suggest that *VDR* overexpression alleviated renal injury and glucose metabolism reprogramming in LPS-induced AKI.

### 3.3 The *VDR* agonist paricalcitol protected against LPS-induced AKI and alleviated glucose metabolism reprogramming

**FIGURE 5:**
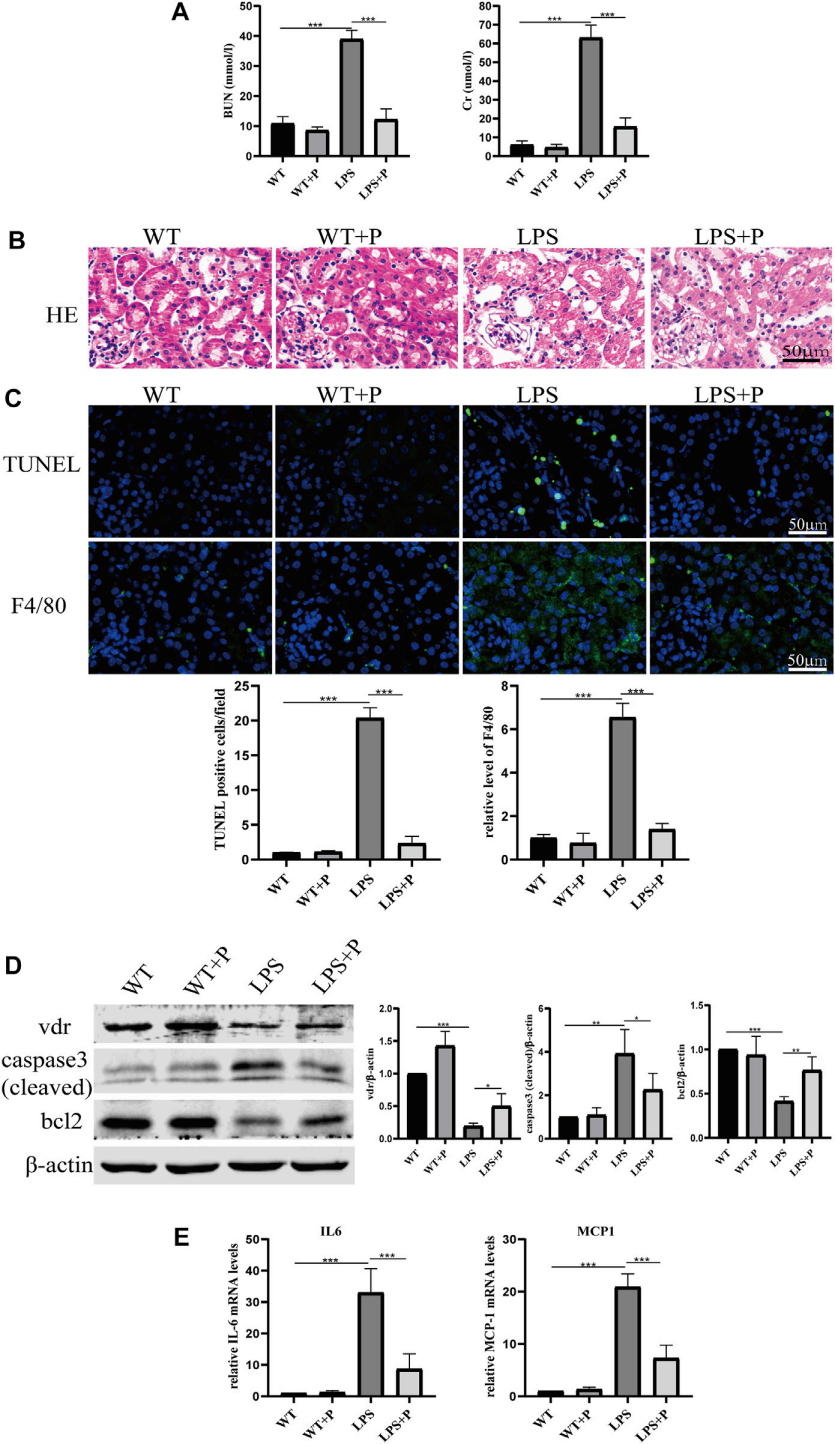
Paricalcitol alleviated renal injury on LPS-induced AKI mice. **(A)** Serum concentrations of BUN and Cr at 24 h after LPS administration. **(B)** H&E staining of kidney sections. **(C)** Immunofluorescence analysis and its quantitative analysis of TUNEL (top) and F4/80 (bottom) of kidney sections. **(D)** Western blot analysis (left) and densitometric quantitation (right) of *VDR*, cleaved caspase3 and bcl2 was performed in the four groups of mice. **(E)** Real-time RT-PCR quantification of IL-6 and MCP1 in the renal cortex of the four groups of mice. *p < 0.05; **p < 0.01; ***p < 0.001. P, Paricalcitol; BUN, blood urea nitrogen; Cr, creatinine; H&E, Hematoxylin and eosin; MCP1, monocyte chemoattractant protein-1.

**FIGURE 6:**
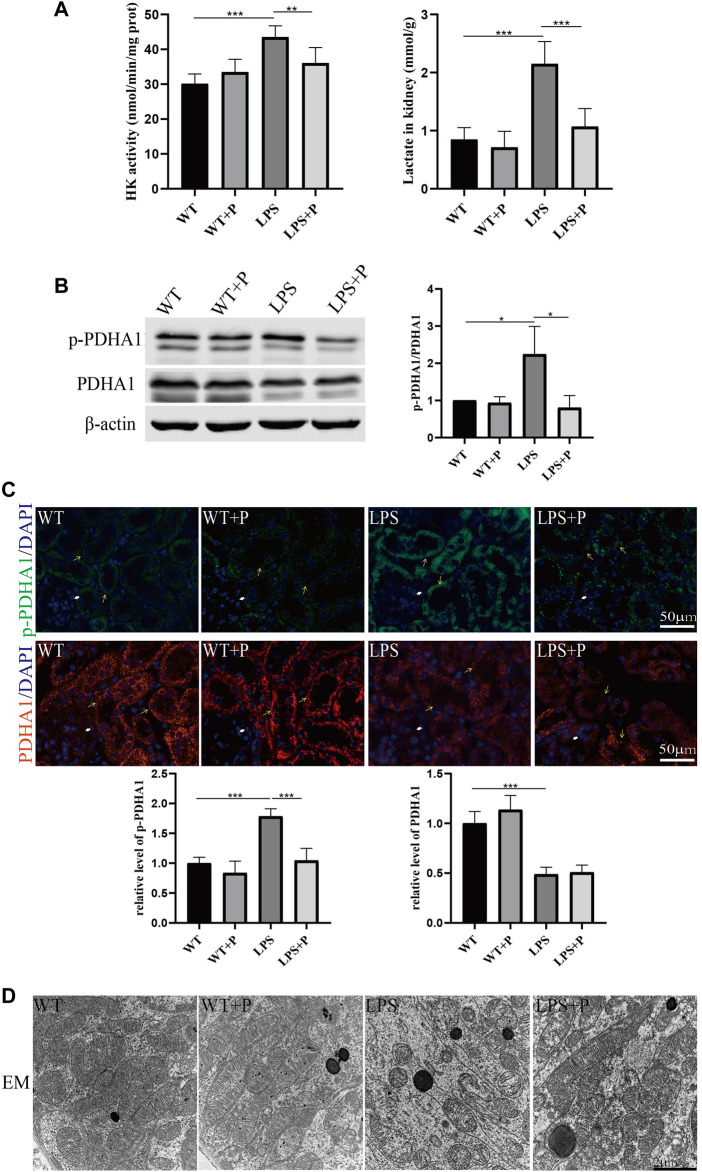
Paricalcitol alleviated glucose metabolism reprogramming of LPS-induced AKI mice. **(A)** Renal lactate content and hexokinase activity of the four groups. **(B)** Western blot analysis (left) and densitometric quantitation (right) of *PDHA1* and p-*PDHA1* and was performed in the four groups of mice. **(C)** Immunofluorescence analysis and its quantitative analysis of p-*PDHA1* (green) and *PDHA1* (red) of kidney sections. White arrow: glomerulus; yellow arrow: renal tubules. **(D)** Images of mitochondrial injury of proximal tubule epithelial cells from the four groups of mice by TEM. *p < 0.05; **p < 0.01; ***p < 0.001.

Paricalcitol (pari), an active vitamin D analog, was used in our study to illustrate the role of vitamin D in LPS-induced AKI. Our results show that pari treatment ameliorated renal insufficiency and pathological damage induced by LPS in C57 mice (Figures 5A, B). Additionally, elevated tubular cell apoptosis and interstitial inflammatory infiltrate in LPS-treated mice were inhibited in the LPS + P group (Figures 5C–E), which is consistent with our previous study (Du et al., 2019). Moreover, the increased lactate accumulation, hexokinase activity and p*-PDHA1*/*PDHA1* ratio in LPS-treated mice were relieved by pari treatment (Figures 6A–C). The mitochondrial damage induced by LPS injection was significantly attenuated by pari treatment (Figure 6D). These results confirm that paricalcitol alleviated glucose metabolism reprogramming in LPS-induced acute kidney injury.

### 3.4 *VDR* alleviates LPS-induced glucose metabolism reprogramming and cell injury in HK2 cells

**FIGURE 7:**
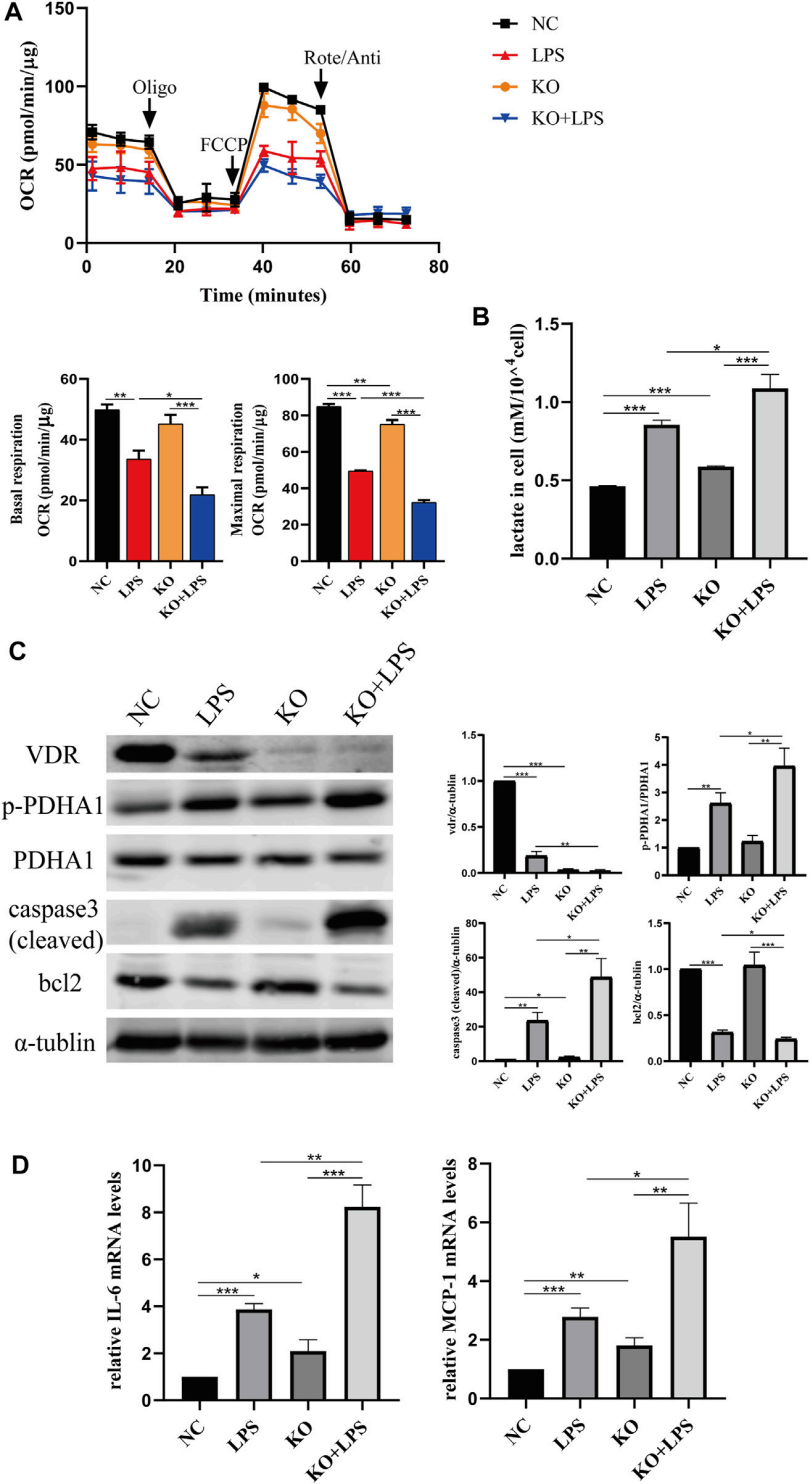
*VDR* deletion aggravated abnormal glycolysis and injury in LPS-induced HK-2 cell. **(A)** Oxygen consumption rate (OCR) (top) measured by Seahorse metabolic analyzer and quantitative analysis (bottom) of mitochondrial function parameters (basal respiration, maximal respiration). **(B)** lactate content in HK-2 cells and *VDR-*KO cells treated with LPS for 24 h. **(C)** Western blot analysis (left) and densitometric quantitation (right) of *VDR, PDHA1*, p-*PDHA1*, cleaved caspase3 and bcl2 was performed in the four groups of HK-2 cells. **(D)** Real-time RT-PCR quantification of IL-6 and MCP1. *p < 0.05; **p < 0.01; ***p < 0.001. Oligo, oligomycin; Rote, rotenone; Anti, antimycin A.

**FIGURE 8:**
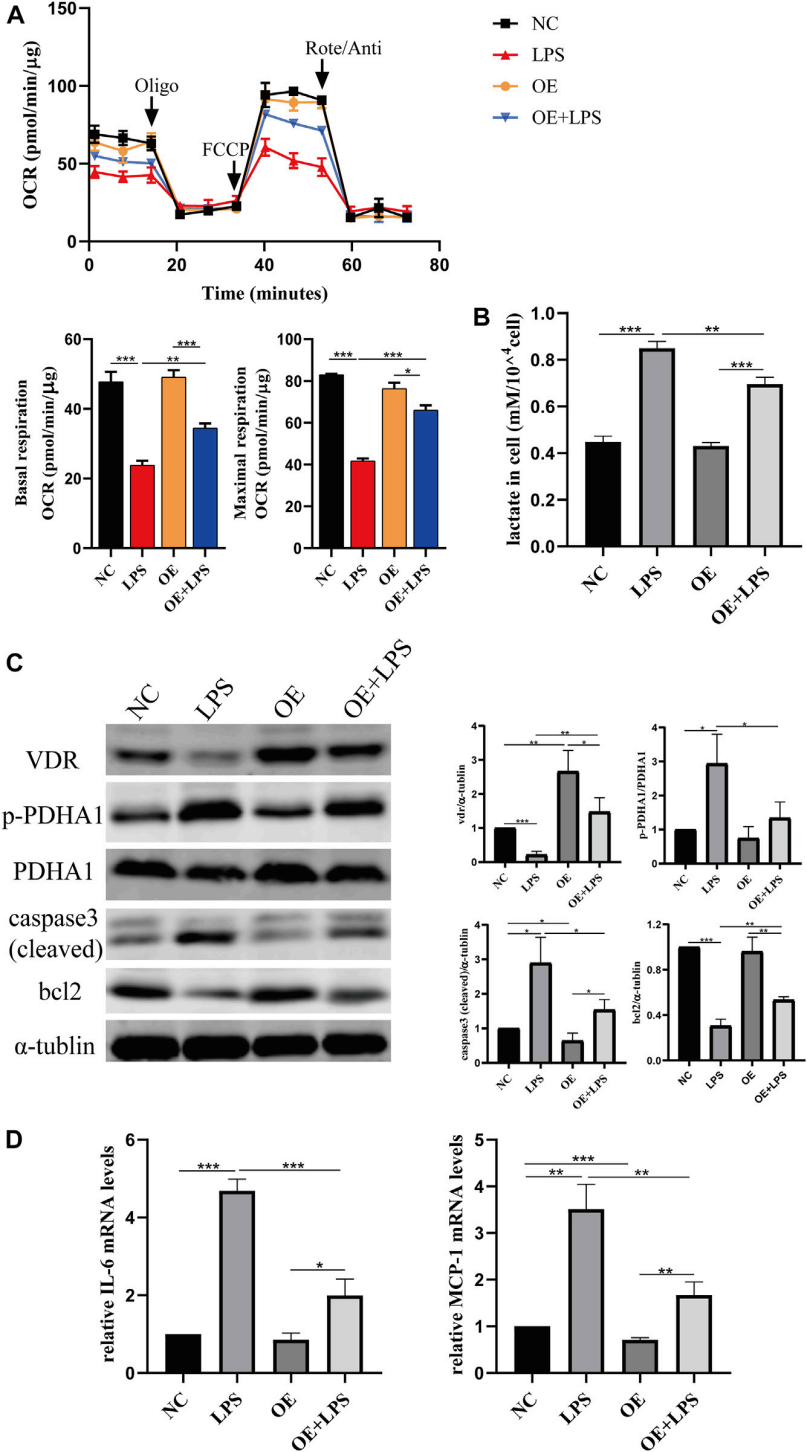
*VDR* overexpression lightened LPS-induced abnormal glycolysis and injury in HK-2 cell. **(A)** Oxygen consumption rate (OCR) (top) measured by Seahorse metabolic analyzer and quantitative analysis (bottom) of mitochondrial function parameters (basal respiration, maximal respiration). **(B)** lactate content in HK-2 cells and *VDR-*OE cells treated with LPS for 24 h. **(C)** Western blot analysis (left) and densitometric quantitation (right) of *VDR, PDHA1*, p-*PDHA1*, cleaved caspase3 and bcl2 was performed in the four groups of HK-2 cells. **(D)** Real-time RT-PCR quantification of IL-6 and MCP1. *p < 0.05; **p < 0.01; ***p < 0.001. Oligo, oligomycin; Rote, rotenone; Anti, antimycin A.

We constructed *VDR* knockout and *VDR* overexpression HK-2 cell lines to verify the effect of *VDR* on LPS-induced glucose metabolism reprogramming *in vitro*. Our results show that LPS induced glucose metabolism reprogramming, including a decreased oxygen consumption rate (OCR) and increased lactate levels, which was more serious in *VDR*-KO cells (Figures 7A, B). On the contrary, overexpression of *VDR* significantly attenuated LPS induced reprogramming of glucose metabolism featured with restored OCR and decreased lactate levels (Figures 8A, B). Furthermore, compared with the LPS group, the KO + LPS group had a higher p-*PDHA1*/*PDHA1* ratio (Figure 7C), while the OE + LPS group had a lower p-*PDHA1*/*PDHA1* ratio (Figure 8C). The expression of caspase-3 (cleaved) and bcl2 by western blot (Figures 7C, 8C) and the mRNA expression of IL-6 and MCP-1 (by PCR) (Figures 7D, 8D) showed that VDR knockout could promote cell apoptosis and inflammation in HK-2 cells, while VDR overexpression could improve these alterations induced by LPS. These results suggests that *VDR* alleviates LPS-induced glucose metabolism reprogramming and cell injury in renal tubular cells.

### 3.5 VD-*VDR* alleviates LPS-induced glucose metabolism reprogramming by inhibiting the phosphorylation of *PDHA1*

**FIGURE 9:**
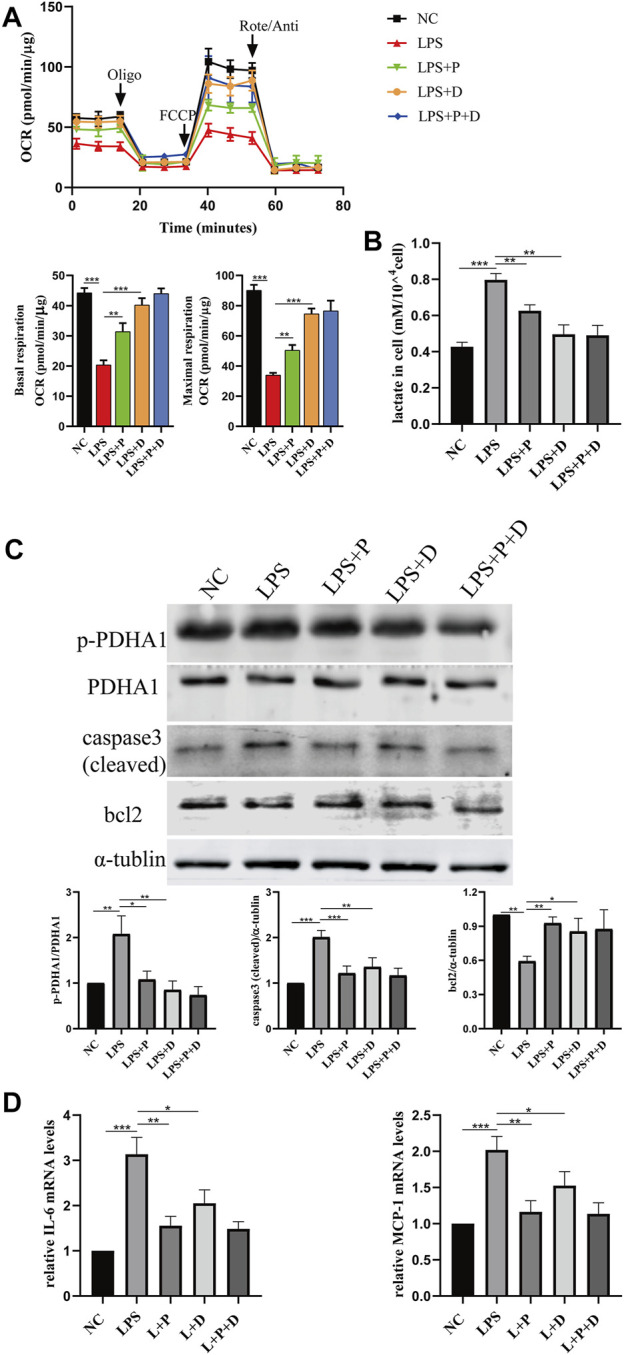
Paricalcitol alleviated LPS-induced injury through phosphorylation of *PDHA1*. **(A)** Oxygen consumption rate (OCR) (top) measured by Seahorse metabolic analyzer and quantitative analysis (bottom) of mitochondrial function parameters (basal respiration, maximal respiration). **(B)** lactate content in HK-2 cells treated with LPS, LPS + P, LPS + D (D: DCA, 5mM, pretreated 2 h), LPS + P + D for 24 h. **(C)** Western blot analysis (top) and densitometric quantitation (bottom) of *PDHA1*, p-*PDHA1*, cleaved caspase3 and bcl2 was performed in the five groups of HK-2 cells. **(D)** Real-time RT-PCR quantification of IL-6 and MCP1. *p < 0.05; **p < 0.01; ***p < 0.001. P, paricalcitol; D: DCA, dichloroacetic acid solution. Oligo, oligomycin; Rote, rotenone; Anti, antimycin A.

The above results indicate that VD-*VDR* can reduce the level of phosphorylated *PDHA1* (p-*PDHA1*) but has no evident effect on the total protein level of *PDHA1* in LPS-induced glucose metabolism reprogramming. Thus, to confirm that *VDR* can play a protective role in glucose metabolism reprogramming and renal injury in LPS-induced AKI by regulating *PDHA1*, DCA (a p-*PDHA1* inhibitor) was used to evaluate LPS-induced tubular cell injury *in vitro*. In HK2 cells, the decreased OCR levels and increased cellular lactate accumulation induced by LPS were protected by paricalcitol or the p-*PDHA1* inhibitor DCA, respectively. Importantly, when treated with both DCA and pari, the protective effect on the glucose metabolism reprogramming of HK-2 cells was no better than that of DCA alone (Figures 9A, B), and the same phenomenon also appeared in the impact on the ratio of p-*PDHA1*/*PDHA1* expression (Figure 9C). Interestingly, the anti-apoptotic and anti-inflammatory effects of the DCA and pari combination in LPS-induced HK2 cells were comparable to those of pari alone (Figures 9C, D). These data confirm that *VDR* activation can alleviate LPS-induced glucose metabolism reprogramming by inhibiting the phosphorylation of *PDHA1*.

### 3.6 VD-*VDR* alleviates glucose metabolism reprogramming *via* the activation of *AMPK* in LPS-induced renal cell injury

**FIGURE 10:**
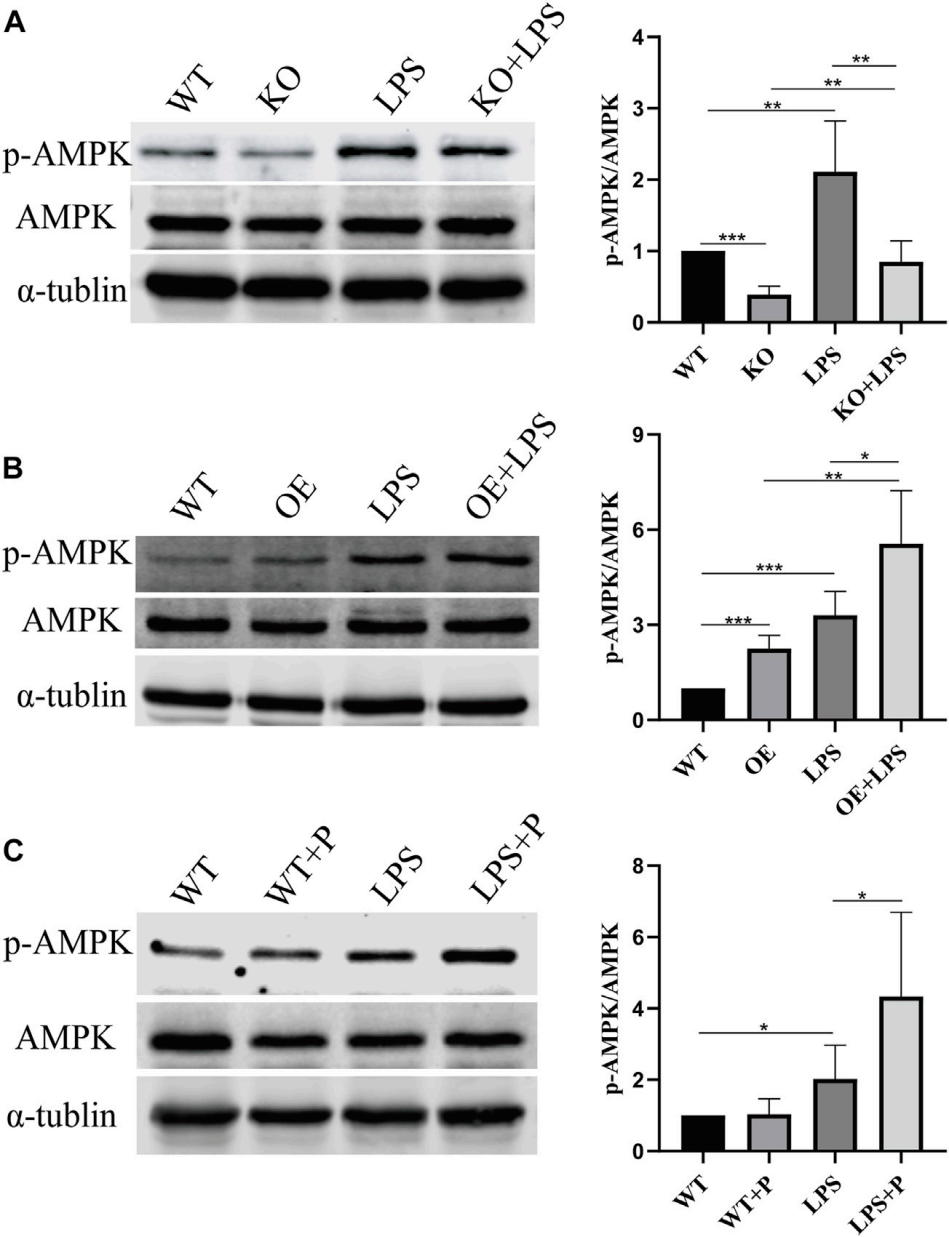
VD-*VDR* active *AMPK* in LPS-induced AKI mice. **(A)** Western blot analysis (left) and densitometric quantitation (right) of *AMPK* and p-*AMPK* was performed in group of WT, KO, LPS and KO + LPS. **(B)** Western blot analysis (left) and densitometric quantitation (right) of *AMPK* and p-*AMPK* was performed in group of WT, OE, LPS and OE + LPS. **(C)** Western blot analysis (left) and densitometric quantitation (right) of *AMPK* and p-*AMPK* was performed in group of WT, WT + P, LPS and LPS + P. *p < 0.05; **p < 0.01; ***p < 0.001.

**FIGURE 11:**
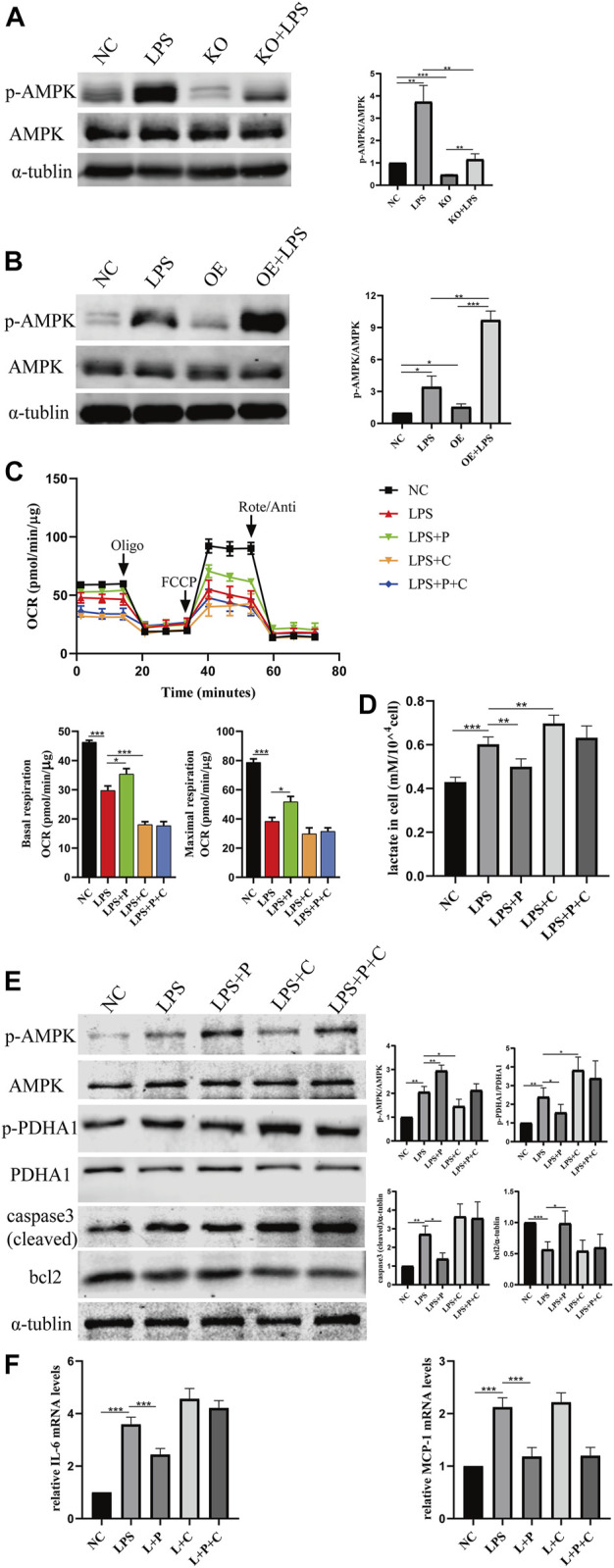
VD alleviate glucose metabolism reprogramming *via* the activation of *AMPK* pathway. **(A)** Western blot analysis (left) and densitometric quantitation (right) of *AMPK* and p-*AMPK in VDR-*KO cells treated with LPS for 24 h. **(B)** Western blot analysis (left) and densitometric quantitation (right) of *AMPK* and p-*AMPK in VDR-*OE cells treated with LPS for 24 h. **(C)** Oxygen consumption rate (OCR) (top) measured by Seahorse metabolic analyzer and quantitative (bottom) analysis of mitochondrial function parameters (basal respiration, maximal respiration). **(D)** lactate content in HK-2 cells treated with LPS, LPS + P, LPS + C (C: compound C, 10 μM, pretreated 1 h), LPS + P + C for 12–16 h. **(E)** Western blot analysis (left) and densitometric quantitation (right) of *AMPK*, p-*AMPK*, *PDHA1*, p-*PDHA1*, cleaved caspase3 and bcl2 was performed in the five groups of HK-2 cells. **(F)** Real-time RT-PCR quantification of IL-6 and MCP1. *p < 0.05; **p < 0.01; ***p < 0.001. Oligo, oligomycin; Rote, rotenone; Anti, antimycin A.

We further investigated how VD-*VDR* inhibits the phosphorylation of *PDHA1*. Since our previous research confirmed that VD-*VDR* can activate *AMPK* in diabetic nephropathy (Li et al., 2022), we detected the level of protein expression of p-*AMPK* in LPS-induced AKI mice and LPS-treated HK-2 cells. As Figures 10, 11 show, p-*AMPK* levels were increased in LPS-induced AKI mice and LPS-treated HK-2 cells, and paricalcitol or *VDR* overexpression further promoted the expression of p-*AMPK* (Figures 10B, C, 11B). In addition, the increased level of p-*AMPK* was weakened in *VDR*-KO mice and HK-2 cells (Figures 10A, 11A).

It has been reported that the glycolysis shift induced by LPS is related to *AMP*-activated protein kinase (*AMPK*) (Tan et al., 2021). Therefore, we examined the protein expression of p-*AMPK*/*AMPK* in LPS-treated HK2 cells and observed glycolytic metabolism in HK2 cells treated with Compound C, an *AMPK* inhibitor. As our data show, Compound C and LPS induced the metabolic state switch from oxidative phosphorylation (lower OCR levels) to glycolysis (higher lactic acid levels), while the effects on alleviating glucose metabolism reprogramming by pari were greatly weakened after Compound C treatment (Figures 11C, D). Similar results were observed in the effect of Compound C on the expression of p-*PDHA1*/*PDHA1* and caspase-3 (cleaved) (Figure 11E). Real-time PCR analysis indicated that Compound C could induce increased expression of IL-6 mRNA, and the reduction in IL-6 by paricalcitol was also largely abolished in the presence of Compound C, but it had no influence on MCP-1 mRNA (Figure 11F).

## 4 Discussion

In this study, we report the role of VD-*VDR* in renal glucose metabolism reprogramming induced by LPS for the first time. Our data show that paricalcitol treatment or *VDR*-specific overexpression restored glucose metabolism reprogramming and renal injury in LPS-induced AKI, whereas *VDR*-KO resulted in a more severe glycolytic shift and renal injury. In addition, we also initially found that paricalcitol attenuated LPS-induced reprogramming of glucose metabolism in HK-2 cells partially *via* the *AMPK* pathway.

Glucose metabolism reprogramming is increasingly recognized as a potentially effective therapeutic strategy for the progression of AKI (Li et al., 2021). Researchers have proposed that metabolic reprogramming exerts renal protection by making up for the damaged energy supply in a short time at the early stage of SA-AKI (Gómez et al., 2017). However, most evidence shows that the aerobic glycolysis transition of renal tubular epithelial cells is harmful and aggravates the damage to renal function. This is because during sepsis, continuous and different injuries may significantly magnify tubular injuries in cells (Toro et al., 2021). In addition, accumulation of lactate and the end product of glycolysis can activate innate immune and inflammatory responses through *TLR*-mediated *NF-κB* signaling and inflammasomes (Samuvel et al., 2009), while the content of pyruvate, which has anti-inflammatory and antioxidant effects, is reduced (Zager et al., 2014), while inflammatory factors are continuously secreted, resulting in a persistent inflammatory state and mitochondrial damage, leading to renal tubular epithelial cell damage. Moreover, the inhibition of aerobic glycolysis alleviates SA-AKI (Tan et al., 2021). Therefore, it is important to reduce glucose metabolic reprogramming to restore renal function in SA-AKI.

Glucose metabolism reprogramming is regulated by many metabolic enzymes. Hexokinase (*HK2*), the first rate-limiting enzyme of glycolytic metabolism, and lactic acid, the final metabolite of glycolysis under anaerobic conditions, both reflect the glycolysis activity. The OCR level and dephosphorylation level of *PDHA1* reflect the aerobic oxidation activity. During SA-AKI, lactate is elevated in septic pigs (Chvojka et al., 2008), and sepsis induces a metabolic shift to aerobic glycolysis in CLP mice (Waltz et al., 2016) and LPS-treated HK-2 cells (Ji et al., 2021). Our data show that LPS-injected mice had a higher lactate concentration and hexokinase activity in renal homogenate than wild-type mice, and LPS-treated HK-2 cells had decreased OCR levels and higher lactate levels than the control group, which is consistent with previously mentioned reports. This runaway reprogramming of glucose metabolism can be restored by paricalcitol, a *VDR* agonist. *VDR* can inhibit glycolysis in colorectal cancer (Zuo et al., 2020), and VD supplementation can improve mitochondrial respiration in primary trophoblasts isolated from obese women (Phillips et al., 2022). Ryan et al. (2016) found that phosphorylated pyruvate dehydrogenase (*PDH*) (Ser-293) decreased in 1α,25(OH)2D3-treated human skeletal muscle cells. However, the effect of VD-*VDR* on the reprogramming of glucose metabolism has not been studied in AKI. Our results show that paricalcitol reduced the elevated lactate concentration and hexokinase activities in LPS-induced AKI mice; more importantly, paricalcitol inhibited the phosphorylation of *PDHA1*. Additionally, we constructed an LPS-AKI model in *VDR*-KO and *VDR*-OE mice to explore the effect of *VDR* on the reprogramming of glucose metabolism in LPS-AKI. As expected, knockout of *VDR* aggravated glucose metabolism reprogramming in LPS-AKI mice, including lactate accumulation and hexokinase activity, whereas overexpression of *VDR* attenuated glucose metabolism reprogramming. Moreover, our *in vitro* experimental data are consistent with those found in animal experiments. These results confirm that VD-*VDR* could alleviate glucose metabolism reprogramming in LPS-induced acute kidney injury.

In our animal experiments, we confirmed the regulatory effect of *VDR* on p-*PDHA1*; however, the specific regulatory mechanism is still unclear. *PDHA1* is a key site regulating *PDH*c, and its phosphorylation is involved in the pathological mechanism of many diseases. Oh et al. found that phosphorylation of *PDHA1* mediates cisplatin-induced acute kidney injury and may be a therapeutic target for cisplatin-induced acute kidney injury (Oh et al., 2017). Pan et al. found that *PDHA1* dephosphorylation reduces pyroptosis-induced inflammation (Pan et al., 2022). In the present work, we used DCA, an inhibitor of p-*PDHA1*, to explore the regulation of p*-PDHA1* by *VDR*. Our results show that in LPS-treated HK2 cells, inhibition of p*-PDHA1* reduces cell glucose metabolic reprogramming, cell inflammation and apoptosis. It seems that the protective effects of the DCA and paricalcitol combination in LPS-induced HK2 cells were comparable to those of paricalcitol alone. This finding supports that paricalcitol can alleviate glucose metabolism reprogramming by inhibiting p*-PDHA1*.

*AMP*-activated protein kinase (*AMPK*) is a classic energy receptor. Under conditions of metabolic stress, such as hypoxia and ischemia, *AMPK* is activated to increase ATP production and reduce ATP consumption to maintain cellular energy homeostasis (Burkewitz et al., 2014; Hardie, 2015). Zhang et al. (2022) showed that *AMPK* antagonizes nickel-refining fume-induced aerobic glycolysis. Similarly, Tang et al. (2021) found that enhanced glycolysis in lung fibroblasts induced by LPS can be prevented by regulating the *AMPK* pathway. Similarly, Tang et al. (2021) found that enhanced glycolysis in lung fibroblasts induced by LPS can be prevented by regulating the *AMPK* pathway (Jin et al., 2020). *AMPK* plays an important role in glucose metabolism reprogramming and is also a fundamental regulator of many pathways involved in energy metabolism (Hardie et al., 2012). Herein, we detected the expression of p*-AMPK*/*AMPK* experimentally and confirmed that the *AMPK* pathway was activated in LPS-induced AKI mice, and the activation of *VDR* could further increase the expression of p-*AMPK*. Notably, the activation of *AMPK* during sepsis is an early adaptive response to injury, while the pharmacological activation of *AMPK* can protect against AKI and improve the survival rate of SA-AKI mice (Jin et al., 2020). In our experiment, VD-*VDR* mediates the activation of the *AMPK* pathway and plays a protective role against AKI, which is consistent with our previous report (Li et al., 2022).

It has been reported that *PDHc* activity can be restored by treatment with an *AMPK* activator (Dugan et al., 2013). Cai et al. (2020) also found that S293 phosphorylation of *PDHA1* was increased in *AMPK* knockout cells, while in *AMPK*-activated cells, S293 phosphorylation of *PDHA1* was decreased and *PDH*c was activated. As expected, our results show that the protective effect of paricalcitol on metabolic reprogramming was weakened by the inhibition of the *AMPK* pathway. This result indicates that the *AMPK* pathway is involved in the regulation of the ratio of p-*PDHA1*/*PDHA1* by *VDR* and exerts a reno-protective effect. The results from the above cellular experiments demonstrate that *VDR* regulates *PDHA1* phosphorylation by activating *AMPK*.

Considering that *VDR* is a nuclear transcription factor, we wondered whether there is transcriptional regulation of *PDHA1* by *VDR*. The results show that the expression of *PDHA1* was only slightly downregulated in the kidney tissue of LPS-injected *VDR*-KO mice, which is inconsistent with the downregulation of *VDR*. However, the overall effect of *VDR* on the inhibition of metabolic reprogramming and renal protection is clearly presented, and we speculate that VD-*VDR* may affect metabolic reprogramming by regulating other molecules, which requires further investigation in the future.

Overall, our data demonstrate that VD-*VDR* could alleviate glucose metabolism reprogramming in lipopolysaccharide-induced acute kidney injury, mediated by activation of the *AMPK* pathway. Our work provides new insight into the renoprotective effect of vitamin D-*VDR* in SA-AKI and provides a promising target for AKI prevention and treatment.

## Data Availability

The raw data supporting the conclusion of this article will be made available by the authors, without undue reservation.
