# Supplementary figures and images for: Vitamin D-VDR (vitamin D receptor) alleviates glucose metabolism reprogramming in lipopolysaccharide-induced acute kidney injury
Source: Front Physiol. 2023 Feb 24;14:1083643. doi: 10.3389/fphys.2023.1083643 (PMC9998528; doi:10.3389/fphys.2023.1083643)

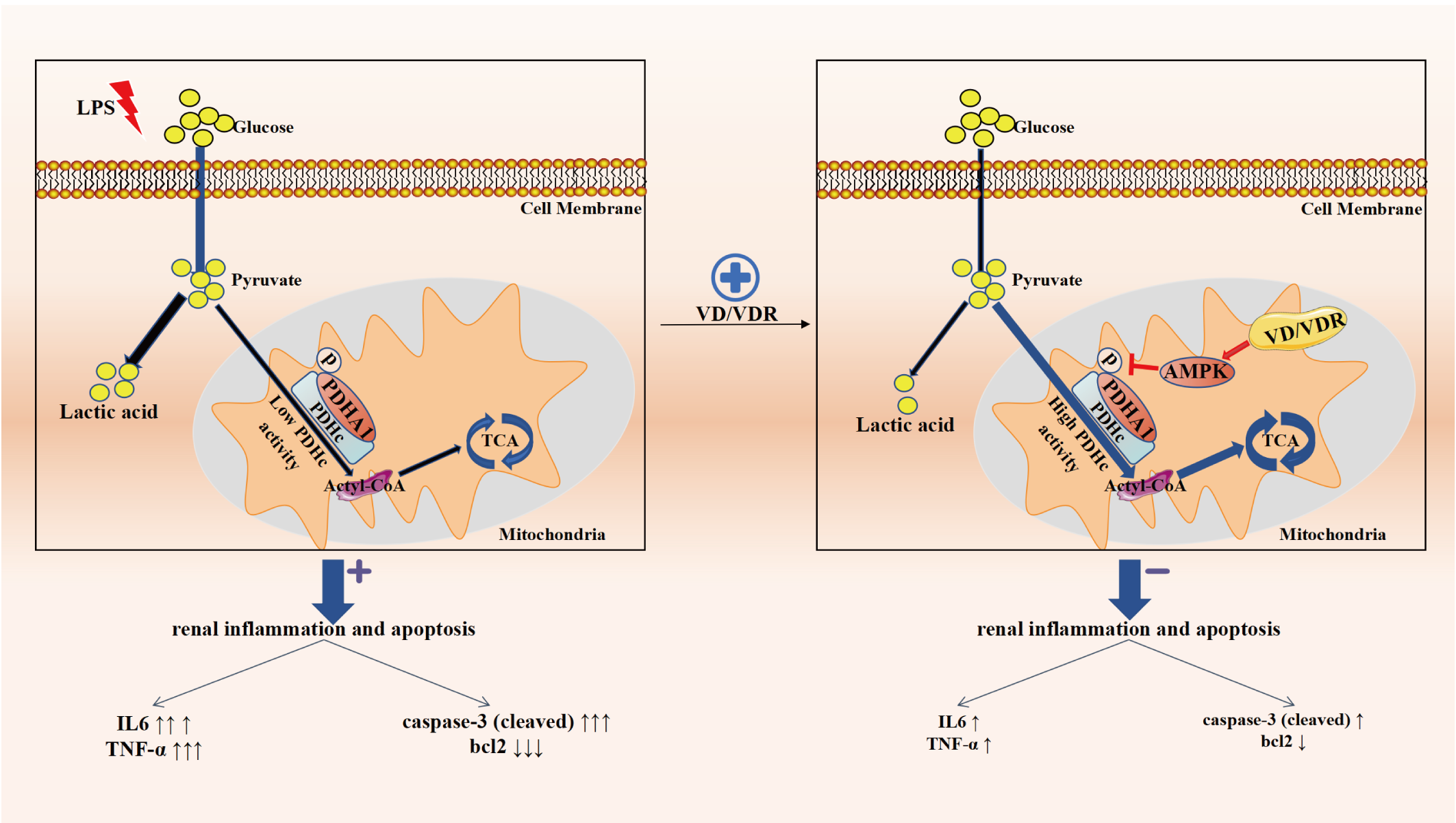

Supplement: Supplementary file 1 [file Image1.TIF]
